# Demarcated thresholds of tumor-specific CD8 T cells elicited by MCMV-based vaccine vectors provide robust correlates of protection

**DOI:** 10.1186/s40425-019-0500-9

**Published:** 2019-01-31

**Authors:** Elham Beyranvand Nejad, Robert B. Ratts, Eleni Panagioti, Christine Meyer, Jennifer D. Oduro, Luka Cicin-Sain, Klaus Früh, Sjoerd H. van der Burg, Ramon Arens

**Affiliations:** 1 Department of Immunohematology and Blood Transfusion, Leiden University Medical Center, Albinusdreef 2, 2333 ZA Leiden, The Netherlands; 2 Department of Medical Oncology, Leiden University Medical Center, Leiden, The Netherlands; 3 Vir Biotechnology, Portland, OR USA; 4 Department of Vaccinology and Applied Microbiology, Helmholtz Centre for Infection Research, Braunschweig, Germany; 5 Institute for Virology, Hannover Medical School, Hannover, Germany; 6 German Centre for Infection Research (DZIF), Partner site, Hannover/Braunschweig, Germany

**Keywords:** T cells, Cancer, CMV-based vaccine vector, Pre-existing immunity

## Abstract

**Background:**

The capacity of cytomegalovirus (CMV) to elicit long-lasting strong T cell responses, and the ability to engineer the genome of this DNA virus positions CMV-based vaccine vectors highly suitable as a cancer vaccine platform. Defined immune thresholds for tumor protection and the factors affecting such thresholds have not well been investigated in cancer immunotherapy. We here determined using CMV as a vaccine platform whether critical thresholds of vaccine-specific T cell responses can be established that relate to tumor protection, and which factors control such thresholds.

**Methods:**

We generated CMV-based vaccine vectors expressing the E7 epitope and tested these in preclinical models of HPV16-induced cancer. Vaccination was applied via different doses and routes (intraperitoneal (IP), subcutaneous (SC) and intranasal (IN)). The magnitude, kinetics and phenotype of the circulating tumor-specific CD8^+^ T cell response were determined. Mice were subsequently challenged with tumor cells, and the tumor protection was monitored.

**Results:**

Immunization with CMV-based vaccines via the IP or SC route eliciting vaccine-induced CD8^+^ T cell responses of > 0.3% of the total circulating CD8 T cell population fully protects mice against lethal tumor challenge. However, low dose inoculations via the IP or SC route or IN vaccination elicited vaccine-induced CD8^+^ T cell responses that did not reach protective thresholds for tumor protection. In addition, whereas weak pre-existing immunity did not alter the protective thresholds of the vaccine-specific T cell response following subsequent immunization with CMV-based vaccine vectors, strong pre-existing immunity inhibited the development of vaccine-induced T cells and their control on tumor progression.

**Conclusions:**

This study highlight the effectiveness of CMV-based vaccine vectors, and shows that demarcated thresholds of vaccine-specific T cells could be defined that correlate to tumor protection. Together, these results may hold importance for cancer vaccine development to achieve high efficacy in vaccine recipients.

**Electronic supplementary material:**

The online version of this article (10.1186/s40425-019-0500-9) contains supplementary material, which is available to authorized users.

## Background

The role of the immune system in cancer eradication has been firmly established. Immunotherapy of cancer has set itself as a mainstream therapy among the conventional therapies comprising chemotherapy, radiotherapy and surgery. Adoptive cell therapy (ACT) with tumor-specific T cells and immunotherapeutic approaches in which the inhibitory pathways are blocked or the costimulatory pathways are triggered to invigorate tumor-specific T cells have shown efficacy in a significant number of patients [1, 2]. Vaccination is another promising form of cancer immunotherapy that has been extensively explored, yet few therapeutic vaccines have shown clinical benefit. The latter has been attributed to the lack of inducing substantial long-lasting functional T cell responses that are able to overcome the immunosuppressive environment [3].

Immune thresholds are instrumental to determine vaccine efficacy and effectiveness against pathogens [4]. In cancer immunotherapy, however, tumor protection thresholds and the factors affecting such thresholds have not well been investigated although as such they are instrumental for both practical reasons and mechanistic insights. To elicit and maintain high immune responses, cytomegalovirus (CMV) is considered as a promising vaccine vector. CMV is unique among viruses as chronic infection by this common betaherpesvirus is characterized by high frequencies of virus-specific T cells and antibodies that do not decline after primary infection but remain high or even increase over time in a process termed memory inflation [5–8]. Inflationary T cells are mostly effector-memory (EM)-like in phenotype, remain polyfunctional for life, and are found in both lymphoid organs and tissues [9]. Despite the induction of host immune responses, CMVs are still able to re-infect [10–12]. Based on these properties, together with the ability to engineer the genome of CMV to attenuate the pathogenicity and to express foreign antigens [13], CMV-based vaccines are currently being explored. In mice and non-human primates, CMV-based vaccines have demonstrated significant protection against pathogens including simian immunodeficiency virus (SIV), mycobacterium tuberculosis and Ebola virus [14–18]. Moreover, CMV-based vaccines comprising tumor antigens, including melanoma antigens gp100 and TRP2 [19–22], human prostate-specific antigen (PSA) [23], or model antigens like ovalbumin [22, 24], have shown anti-tumor efficacy in prophylactic and therapeutic mouse models.

Here we studied whether thresholds of vaccine-elicited T cell responses could be determined that relate to the efficacy of such vaccines to protect against tumor progression, and which factors influence these thresholds. Specifically, we investigated the vaccine thresholds of CMV-based vaccine vectors and the impact of inoculum dosages and routes of infection to achieve tumor protection. Given the high world-wide CMV prevalence, and the fact that pre-existing immunity varies, as evidenced by large variations in the magnitude of CMV-specific T cell responses in the population, ranging from barely detectable to 40% of the memory compartment, we also addressed the impact of pre-existing immunity on the protective thresholds [7]. Defining protection thresholds could be highly relevant in determining correlates of protection, considered as a crucial element in vaccine development as it provides the level of vaccine efficacy in vaccine recipients.

We found that CMV-based vaccines eliciting clearly discernible CD8^+^ T cell responses in the blood (> 0.3% of the total CD8 T cell population) fully protected mice against lethal tumor challenge while relatively low responses do not provide protection. Previous exposure to CMV resulting in a weak immune response did not impact vaccine efficacy, however, strong pre-existing immunity hampered the induction of vaccine-induced T cells consequently resulting in loss of tumor protection. The results highlight the premise of CMV-based vaccines, and put forward the potential of such vaccines in halting tumor development based on correlates of protection that are established on careful determination of vaccine-induced immune responses.

## Methods

### Mice

C57BL/6 mice were obtained from Charles River Laboratories (L’Arbresle, France) or Jackson Laboratory (Sacramento, CA, USA). At the start of the experiments, mice were 6 to 8 weeks old. Mice were housed in individually ventilated cages (IVC) under specific pathogen-free (SPF) conditions in the animal facility of Leiden University Medical Centre (LUMC, The Netherlands) or Oregon Health and Science University (OHSU, Oregon, USA). All animal experiments were approved by the institutional Animal Experiments Committee and were executed according to the institutional animal experimentation guidelines and were in compliance with the guidelines of the Institutional, European and Federal USA animal care and usage committees.

### Virus preparation

Viral titers of virus stocks or infected tissues were determined by plaque assays as previously described [25]. MCMV-IE2-E7 (RAHYNIVTF) and MCMV-IE2-OVA (SIINFEKL) were generated as described [26]. MCMV-IE2-E6/E7 full length fusion, MCMV-IE2-E6/E7 full length replace, MCMV-M79-FKBP-E7 (E7 epitope RAHYNIVTF, IE2 fusion), MCMV-M79-FKBP-gB (gB epitope SSIEFARL, IE2 fusion) were constructed as follows. MCMV BACs were maintained in SW105 *E. coli* and modified by metabolic galactokinase (*galK)* selection [27], essentially as described [28]. In brief, bacteria containing the MCMV backbone were first transformed with a PCR product of *galK*/kanamycin (*kan*) and flanking homology to target a specific region in the MCMV BAC. Transformed bacteria were selected for *kan* resistance, grown and subjected to a second transformation to replace the *galK/kan* cassette with the final insert. These included annealed oligos used for IE2 peptide fusion or the PCR product for M79-FKBP [29] and HPV E6/E7 insertions containing the desired modification with the same MCMV flanking homology to insert the *galK/kan* cassette. Recombinant bacteria were counter-selected on chloramphenicol 2-deoxy-galactose (DOG) minimal media plates with glycerol as the carbon source. MCMV BAC constructs were characterized by restriction digest, PCR screening, and Sanger and NGS sequencing. Virus was reconstituted by either Lipofectamine 3000 (ThermoFisher Scientific) transfection or electroporation (250 V and 950 uF) of NIH 3T3s.

Tissue culture-derived stocks of the MCMV vectors were amplified and titered in NIH 3 T3 cells grown in complete growth media (DMEM, FBS, PSG). FKBP-tagged viruses were grown in complete growth media supplemented with Shield-1 at a final concentration of 1 uM and added every 48 h [29]. Cell free virus was obtained from supernatant of infected cells, clarified at 3.000 rpm for 20 min and virus was pelleted at 24.000 rpm for 1 h through a sorbitol cushion (10% D-sorbitol, 0.05 M Tris pH 7.4, 1 mM MgCl_2_). Virus pellet was resuspended in PBS. For virus quantification, plaque assays were performed in 24-well plates by infection with appropriate serial virus dilution in 0.2 mL of media and then incubated at 37 °C for 2 h rocking. Following incubation, the infected cells were overlaid with 1 mL complete media supplemented with carboxymethylcellulose. After 5 to 6 days, the cells were fixed in 3.7% formaldehyde in PBS and stained with 0.001% aqueous methylene blue. The plaques were counted by light microscopy. Multi-step virus replication curves were performed in NIH 3 T3 cells at MOI 0.1 in 6 well plates, 3 replicates per virus per time-point. Virus was incubated at 37 °C for 2 h, washed 3 times with PBS and then 2 mL of media was added. Supernatant was harvested at 1, 3, 5, and 7 days post-infection, stored at − 80 °C and titered by plaque assay. FKBP-tagged viruses were grown in complete growth media supplemented with Shield-1 at a final concentration of 1 uM and added every 48 h.

### Tumor challenge models and anti-tumor vaccination

The tumor cell line TC-1 (a kind gift from T.C. Wu, John Hopkins University, Baltimore, MD) was generated by retroviral transduction of C57BL/6 lung epithelial cells with the HPV16 E6/E7 and c-H-ras oncogenes [30] and cultured as previously described [31]. The tumor cell line C3 was developed by transfection of mouse embryonic cells with the HPV16 genome and an activated-ras oncogene and maintained as previously described [32]. The MC38-OVA tumor cell line is generated by a retroviral infection of the MC38 parental cell-line with PMIG/MSCV-IRES-GFP plasmid encoding cytoplasmic bound OVA [33]. Iscove’s Modified Dulbecco’s Media (IMDM) (Lonza, Basel, Switzerland) supplemented with 8% fetal calf serum (FCS) (Greiner), 2 mM L-glutamine (Life Technologies, Carlsbad, CA, Unites States), 50 IU/ml Penicillin (Life Technologies) and 50 μg/ml Streptomycin (Life Technologies) was used to culture tumor cell lines. Cells were cultured in a humidified incubator at 37 °C and 5% CO_2_. *Mycoplasma* tests that were frequently performed for all cell lines by PCR were negative.

Treatment schedule of experiments are indicated in the respective figures and legends. Mice were vaccinated with MCMV vectors via the intraperitoneal (IP), intranasal (IN) or subcutaneous (SC) route with the indicated inoculum size. In tumor experiments, mice were inoculated subcutaneously in the flank with 0.25–1 × 10^5^ TC-1 tumor cells, 5 × 10^5^ C3 tumor cells or with 2.5 × 10^5^ MC38-OVA in 200 μl PBS containing 0.2% BSA on day 0. Tumor size was measured two times a week using a caliper. Mice were euthanized when tumor size reached > 1000 mm^3^ in volume or when mice lost over > 20% of their total body weight (relative to initial body mass).

### In vivo antibody usage

CD8 T cell depleting monoclonal antibodies (clone 2.43) were purchased from Bio-X-Cell (West Lebanon, NH, United States) and administered IP twice weekly (200 μg/mouse) for 2–3 weeks. CD8 T cell depletion was started 4 days before tumor challenge. Depletion was checked by staining for CD3 and CD8 marker expression followed by flow cytometric analysis.

### Flow cytometry

Blood collection and processing was performed as described [34]. Cells were re-suspended in staining buffer (PBS + 2% FCS + 0.05% sodium azide) and incubated with various fluorescently labelled antibodies detecting CD8 (clone 53–6.7), CD62L (clone MEL-14), CD44 (clone IM7), KLRG1 (clone 2F1), CD3 (clone 500A2), CD127 (clone A7R34). Antibodies were obtained from eBioscience (San Diego, CA, United States), BD Biosciences (San Jose, CA, United States) and Biolegend (San Diego, CA, United States). For dead cell exclusion, 7-Aminoactinomycin D (Invitrogen, Carlsbad, CA, United States) was used. To measure the MCMV-specific and tumor antigen-specific T cell responses, PE and APC-labelled class I-restricted multimers (tetramers or dextramers) with the peptide epitopes OVA_257–264_ (SIINFEKL), HPV E7_49–57_ (RAHYNIVTF), MCMV M45_985–993_ (HGIRNASFI), MCMV M38_316–323_ (SSPPMFRV) were used. Tetramers were produced as described [35] and dextramers were obtained from Immudex. Samples were analyzed with a BD LSRII or LSRFortessa flow cytometer, and results were analyzed using FlowJo software (Tree Star, Ashland, OR, United States).

### In vivo cytotoxicity assay

Splenocytes of naïve CD45.1 (Ly5.1) mice were isolated and loaded with MHC class I restricted-E7_49–57_ or OVA_257–264_ peptides for 1 h at 37 °C at the final concentration of 1 μg/ml. After extensive washing, cells which were pulsed with specific peptide or irrelevant peptide were labelled with high and low concentrations of CFSE (Invitrogen), respectively. Next, 5 × 10^6^ peptide pulsed cells were pooled and injected intravenous (IV) via the retro-orbital route into the mice that have been vaccinated 70 days earlier with MCMV-based vaccines. After 24 h, spleens of the recipient mice were isolated, stained for CD45.1, and subjected to flow cytometry. The cytotoxic capacity was calculated relative to naïve mice by using the following formula: [100 – ((percentage of pulsed peptide in infected mice/percentage of unpulsed peptide in infected mice)/(percentage of pulsed peptide in naive mice/percentage of unpulsed peptide in naive mice)] × 100.

### Enzyme-linked ImmunoSpot (ELISpot) assay

Splenocytes of individual vaccinated mice were isolated and cultured on ELISpot plates (R&D systems, Minneapolis MN) pre-coated with anti-IFN-γ antibodies. The indicated peptides (E7_49–57_, RAHYNIVTF; or a pool of peptides spanning the E7 sequence, 15-mers overlapping by 11) were added at 1 μg/ml. 36 h later cells were removed and secondary antibodies added. Plates were developed according to the manufactures instructions. Spots were counted using an AID ELISpot reader.

### Statistical analysis

Statistical analyses were performed using GraphPad Prism (La Jolla, CA, Unites States). Survival data were analyzed by Kaplan-Meier and the log-rank (Mantel-Cox) test. Statistical significance was determined by Mann Whitney. *P*-values of ≤0.05 were considered statistically significant.

## Results

### Location and size of foreign sequences in CMV-based vaccine vectors impact vaccine-elicited T cell responses and associated tumor protection

**Fig. 1:**
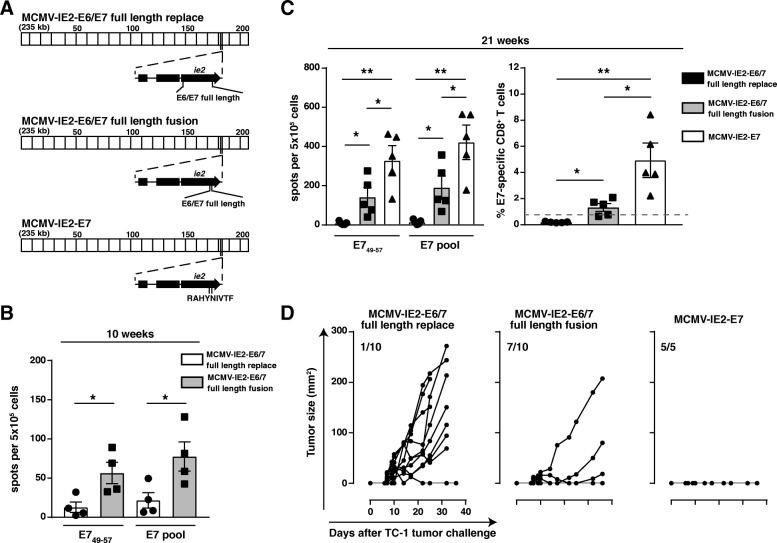
Position and epitope context of heterologous antigens in MCMV-vectored vaccines impact vaccine-elicited T cell responses and associated tumor protection. **a** Schematic of MCMV-IE2-E6/E7 full length replace/fusion and MCV-IE2-E7. The MCMV genome region at ∼kb 186–187 corresponds to the MCMV gene *ie2* (enlarged). Constructs encoding the E6/E7 fusion protein or the E7 epitope were generated by BAC recombineering. DNA sequences encoding the full-length E6/E7 fusion protein were inserted either to replace the *ie2* gene (full length replace) or in frame at the very end of the *ie2* gene (full length fusion). The E7_49–57_ epitope RAHYNIVTF was fused to the end of *ie2*. **b**, **c** C57BL/6 mice were vaccinated IP with 1 × 10^5^ PFU MCMV-IE2-E6/7 full length fusion, MCMV-IE2-E6/7 full length replace or MCMV-IE2-E7. Splenocytes were harvested 10 (**b**) or 21 (**c**) weeks after vaccination and stimulated with the immunodominant E7_49–57_ peptide or with a pool of 15-mer peptides covering the entire E7 protein for 36 h. IFN-γ production was measured by ELISpot assay. Shown is the number of spots per 5 × 10^5^ cells ± SEM. **c** Frequency of E7_49–57_-specific T cells identified by MHC class I multimers within the total CD8^+^ cell population at 21 weeks post vaccination. Data represents mean values ± SEM (*n* = 4–5 mice per group). *, *P* < 0.05; **, *P* < 0.01. **d** Tumor outgrowth of TC-1 tumor cells in mice challenged with TC-1 tumor cells 21 weeks after vaccination with 1 × 10^5^ PFU MCMV-IE2-E6/7 full length replace, MCMV-IE2-E6/7 full length fusion or MCMV-IE2-E7 (5–10 mice per group). The number of tumor-free/total mice is indicated in each tumor out growth graph

To determine effective regions to insert foreign sequences, we generated recombinant MCMV-based vaccine vectors containing the entire E6 and E7 antigens of human papilloma virus type 16 (HPV16). In these vectors the E6/E7 genes replaced exon 3 of IE2 (MCMV-IE2-E6/E7 full length replace) or E6/E7 was placed in the end part of the IE2 region (MCMV-IE2 E6/E7 full length fusion) (Fig. 1a). To analyze the T cell responsiveness to the inserted foreign sequences induced by these vaccine vectors, we IP vaccinated mice and measured the E7-specific T cell responses by IFN-γ ELISPOT, following restimulation with the immunodominant peptide epitope E7_49–57_ or with a pool of overlapping peptides covering the whole E7 protein, and by staining with MHC class I multimers. Immunization with MCMV-IE2-E6/E7 full length fusion induced significantly higher E7-specific T cell responses (~ 0.5–2% of the total CD8^+^ T cell population) than MCMV-IE2-E6/E7 full length replace (< 0.3%) at 10 and 21 weeks after vaccination (Fig. 1b and c). To assess the anti-tumor immunity induced by these recombinant viruses we challenged the vaccinated mice with TC-1 tumor cells, which are transformed by expression of the HPV16 E6 and E7 oncoproteins [30]. Vaccination with MCMV-IE2-E6/E7 full length fusion prevented tumor progression in most tumor-challenged mice, while the majority of the mice vaccinated with MCMV-IE2-E6/E7 full length replace developed tumors (Fig. 1d). Thus, consistent with our previous report we found that fusion of foreign sequences to the C-terminus of the IE2 protein works superior in eliciting T cell responses, and this event relates to higher amounts of the peptide epitope presented in peptide-MHC complexes [26].

Next, we analyzed the impact of the size of the E6/E7 antigens, and therefore generated a recombinant MCMV expressing only a single immunodominant CD8 T cell epitope, i.e. RAHYNIVTF (E7_49–57_), fused to the carboxyl terminus of the MCMV IE2 gene (MCMV-IE2-E7). Clearly, mice immunized with MCMV-IE2-E7 resulted in the highest E7-specific T cell response (~ 2–8%), and vaccination with MCMV-IE2-E7 fully protected mice against otherwise lethal TC-1 tumor challenge (Fig. 1c and d). In conclusion, both the location and size of inserted foreign sequences impact the vaccine-elicited T cell response of MCMV vaccine vectors and this corresponds to protection against tumor outgrowth.

### The magnitude of inflationary T cell responses elicited by recombinant MCMV-based vaccine vectors is influenced by the inoculation route and dose and determines vaccine efficacy

**Fig. 2:**
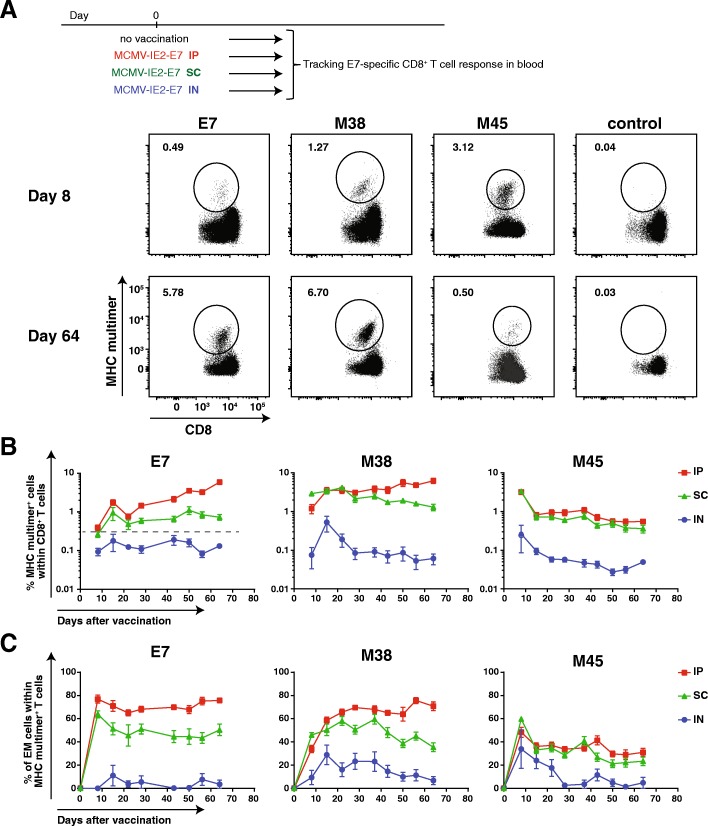
Vaccination with recombinant MCMV vectors via the IP and SC route induces potent specific T cell responses. Mice were vaccinated with MCMV-IE2-E7 via IP, SC or IN route or kept unvaccinated (control), and the CD8 T cell responses were examined in blood. **a** Representative flow cytometry plots for MHC class I multimer staining to E7, M38 and M45 epitopes in blood of MCMV-IE2-E7 infected mice via IP during acute phase (day 8 post vaccination) and memory phase (day 64 post vaccination). Numbers represent the percentage of Ag-specific CD8^+^ T cells within the total CD8^+^ T cell population. Control flow cytometry plots show the staining for E7 multimer in unvaccinated mice. Flow cytometry plots show similar numbers of total cells in each plot except for control. **b** Frequency of Ag-specific CD8^+^ T cells to E7, M38 and M45 epitopes over time. **c** Frequency of effector-memory (EM) cells (KLRG1^+^, CD62L^−^) within E7, M38 and M45 MHC multimer^+^ CD8^+^ T cells over time. Data represents mean values ± SEM (*n* = 7–8 mice per group) and are representative of two independent experiments with similar results

To investigate the impact of the route of inoculation on the application of MCMV-based vaccine vectors for vaccine-elicited immunity against cancer, we compared the magnitude and kinetics of the antigen-specific CD8^+^ T cell response in the blood upon IP, IN and SC inoculation. The E7-specific CD8^+^ T cell responses elicited with MCMV-IE2-E7 increased gradually to ~ 5% after IP immunization, while the responses were steadily ~ 0.6% after SC inoculation. Upon IN inoculation however, the E7-specific CD8^+^ T cell responses were minute throughout infection (< 0.3%) (Fig. 2a and b). The M38_316–323_ epitope-specific CD8^+^ T cell response after IP and SC inoculation showed similar magnitude and kinetics as the response to the E7 epitope in the same vector (Fig. 2a and b). Upon IN immunization, the M38-specific CD8^+^ T cell response was barely detectable and did not show signs of inflation. Upon immunizations via the IP and SC routes, the non-inflationary response to the M45_985–993_ epitope showed the typical response pattern of rapid expansion followed by swift contraction and stable memory formation (Fig. 2a and b). IN vaccination induced only ~ 0.1% M45-specific CD8^+^ T cells, and during the memory phase this response was close to the detection limit (Fig. 2a and b).

Characteristic for inflationary T cells is their effector-memory (EM) like (CD44^+^CD62L^low^, CD127^+/−^, KLRG1^+^) appearance [6]. Early after IP and SC immunization, the E7-specific CD8^+^ T cell population acquired an EM-like phenotypic profile, which remained high throughout the memory phase (Fig. 2c). The M38-specific CD8^+^ T cells displayed a gradual increase of the EM phenotype to 70–80% of the total Ag-specific T cell population. In contrast, only 40% of the M45-specific CD8^+^ T cells showed an EM phenotype throughout the memory phase. Upon IN immunization, the formation of the EM phenotype by the E7, M38 and M45-specific CD8^+^ T cells was much less pronounced (Fig. 2c). Taken together, these data show that immunization with recombinant MCMV vectors via the IP and SC route induces prominent EM-like CD8^+^ T cell responses, albeit with different kinetics, while IN infection resulted in weak responses at best.

**Fig. 3:**
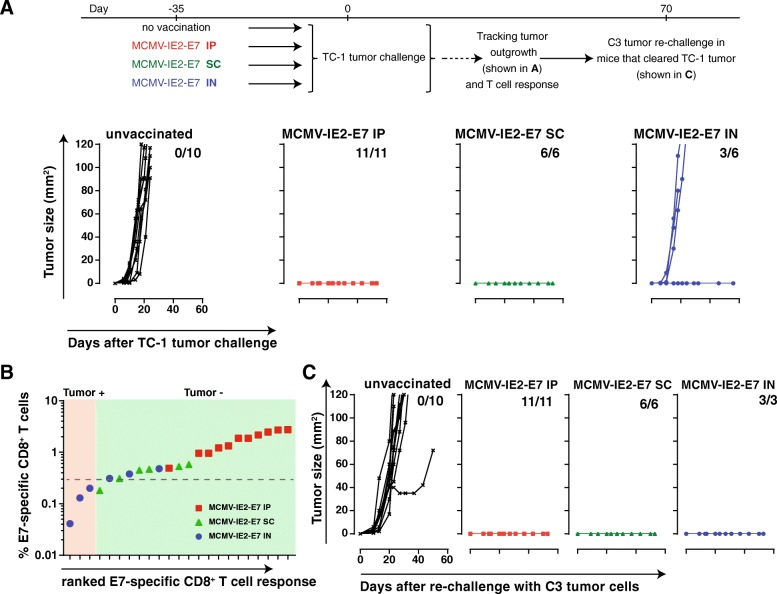
Immunization with MCMV-based vaccine vectors that induce substantial tumor-specific T cell responses via different routes of infection control tumor outgrowth. **a** TC-1 tumor outgrowth of unvaccinated mice and of mice previously vaccinated with MCMV-IE2-E7 via the IP, SC or IN route. The number of tumor-free/total mice is indicated in each graph. **b** The percentage of E7-specific CD8^+^ T cells within the total CD8^+^ T cell population in the blood (y-axis) plotted against the E7-specific CD8^+^ T cell response ranked from low to high (x-axis) of the mice that were either tumor-free (tumor-) or tumor positive (tumor+). **c** The mice that cleared the TC-1 tumor in A were re-challenged with 1 × 10^5^ C3 tumor cells. The number of tumor-free/total mice is indicated in each graph. Data are representative of two independent experiments with similar results

Next, we investigated the influence of the route of immunization and the resulting vaccine-specific T cell response elicited by the MCMV vectors on efficacy in tumor challenge experiments. We vaccinated mice with MCMV-IE2-E7 via the IP, IN or SC routes and challenged these mice with TC-1 tumor cells. While TC-1 tumors grew out progressively in all naïve mice, immunization with MCMV-IE2-E7 via the IP and SC route induced complete protection against TC-1 tumor challenge (Fig. 3a). Conversely, IN immunization with MCMV-IE2-E7 protected only 50% of the mice (Fig. 3a). Notably, the IN immunized mice that were protected had stronger E7-specific CD8^+^ T cell responses as compared to the unprotected group (Fig. 3b). Thus, MCMV vectors inducing circulating tumor-specific CD8^+^ T cell responses > 0.3% provides full protection while responses below this threshold results in loss of protection. Immunizations with MCMV-based vaccine vectors via routes of vaccination that induce large tumor-specific T cell responses are thus superior in controlling tumor outgrowth. In addition, all the mice which remained tumor free until day 60, including the subset of the IN vaccinated mice, were protected against challenge with C3 tumor cells expressing HPV antigens including the E7 oncoprotein (Fig. 3c). Protection against MC38 tumor cells, which do not express E7, was however not established (data not shown). These results also indicate the induction of immunological memory including in those mice vaccinated via the IN route.

To assess the capacity of CMV-based vaccines to elicit responses to other inserted foreign epitopes, we tested MCMV expressing the H-2Kb-restricted OVA_257–264_ (SIINFEKL) epitope from ovalbumin (OVA). Immunization with MCMV-IE2-OVA induced OVA-specific CD8^+^ T cell responses against OVA_257–264_ that were slightly higher than the response against E7 after MCMV-IE2-E7 immunization. The responses against the epitopes in M38 and M45 were similar (Additional file 1: Figure S1A). Moreover, vaccination with MCMV-IE2-OVA induced complete protection against OVA-expressing MC38 tumor cells (Additional file 1: Figure S1B).

**Fig. 4:**
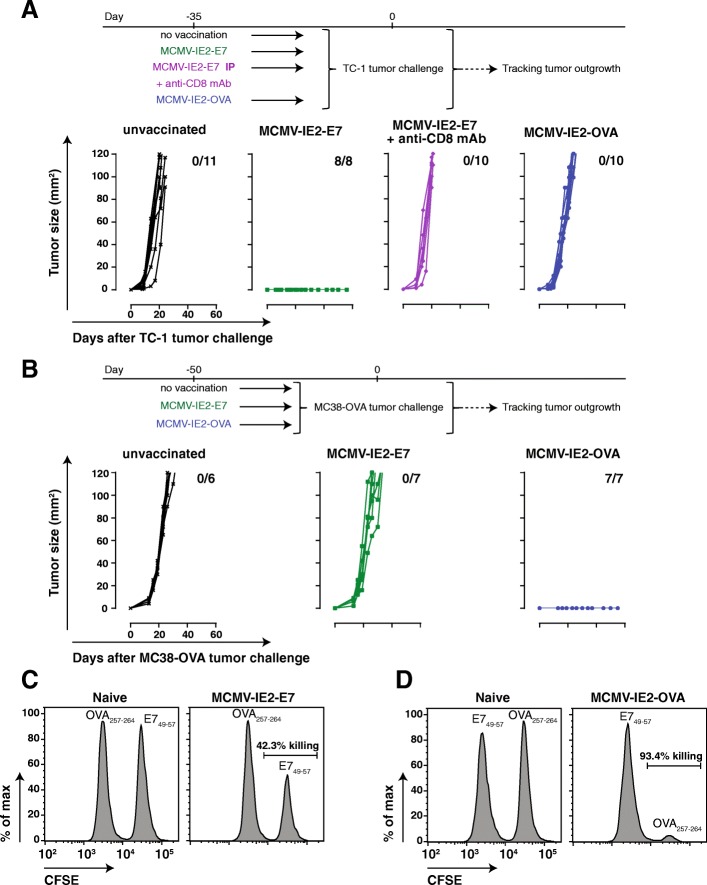
Specific anti-tumor immunity elicited by MCMV-based vaccine vectors is essential for protection. **a** TC-1 tumor outgrowth of unvaccinated mice and of mice previously vaccinated with MCMV-IE2-E7 or MCMV-IE2-OVA via the IP route. CD8 depleting antibody was given in the group of mice vaccinated with MCMV-IE2-E7 via IP starting 4 days before tumor challenge. The number of tumor-free/total mice is indicated in each graph. Data was pooled from two independent experiments. **b** MC38-OVA tumor outgrowth of unvaccinated mice and of mice previously vaccinated with MCMV-IE2-E7 or MCMV-IE2-OVA via the IP route. The number of tumor-free/total mice is indicated in each graph. Data are representative of two independent experiments with similar results. **c** and **d** Representative histograms of CTL-mediated killing responses induced by MCMV-IE2-E7 (**c**) or MCMV-IE2-OVA (**d**). Mice were vaccinated with 5 × 10^5^ PFU MCMV-IE2-E7 (**c**) or MCMV-IE2-OVA (**d**) via SC. After 69 days, splenocytes from naïve mouse were pulsed with specific peptide; E7_49–57_ (**c**) or OVA_257–264_ (**d**) or unspecific peptide and labelled with high and low concentrations of CFSE, respectively. Then, cells were adoptively transferred to the MCMV vaccinated mice or naïve mice as control. One day later, spleens of the mice were analyzed for the percentage of killing as described in materials and methods

Next, we examined whether tumor protection was solely due to the induction of tumor-specific T cell responses or whether possible bystander effects of virus infection were implicated. First, we established the requirement of CD8^+^ T cells in the tumor protective role of MCMV-based vaccine vectors. CD8^+^ T cell depletion 4 days before TC-1 tumor challenge in mice immunized earlier with MCMV-IE2-E7 via the IP route resulted in tumor progression, which underscores the importance of CD8^+^ T cells (Fig. 4a). Second, mice inoculated with MCMV-IE2-OVA and challenged with TC-1 tumor cells, which do not express OVA antigen, were not protected whereas MCMV-IE2-OVA immunization protects against MC38-OVA **(**Fig. 4a and b**)**. Vice versa, MCMV-IE2-E7 immunization does not provide protection against MC38-OVA **(**Fig. 4b**)**, while this vaccine vector provides protection against E7-expressing TC-1 tumor cells **(**Figs. 3a, 4a**)**. To further demonstrate specific target-mediated killing induced by the MCMV-based vaccine vectors, we performed in vivo cytotoxicity assays. Target cells pulsed with vaccine-specific antigen or control antigen were injected into mice previously vaccinated with MCMV-IE2-E7 (Fig. 4c) or MCMV-IE2-OVA (Fig. 4d). Both MCMV-based vaccine vectors induced exclusive killing of target cells presenting the vaccine-specific antigen. Thus, vaccine-induced specific CD8^+^ T cell responses against antigens expressed by the tumor are required to induce protective immunity and responses against the vector alone do not provide protection.

**Fig. 5:**
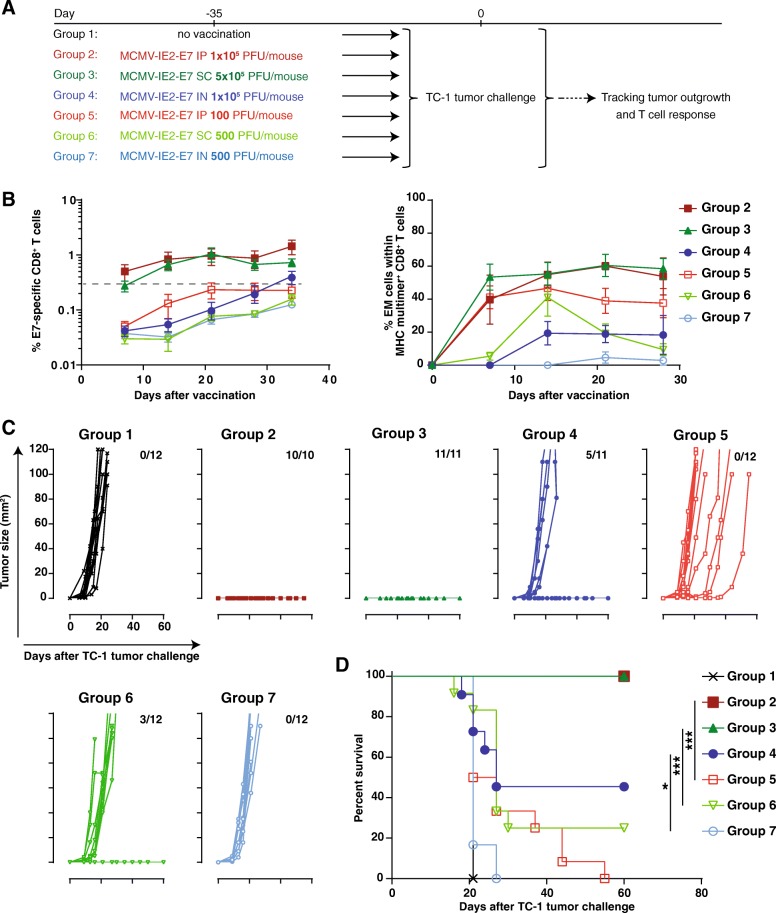
The inoculum dosage of recombinant MCMV-based vaccine vectors determines the magnitude of anti-tumor T cell response and protection efficacy. **a** Schematic of the experiment. Mice were infected with indicated doses and routes or kept unvaccinated. After 35 days, all the mice were challenged with TC-1 tumor cells. The tumor outgrowth and survival of the mice was followed for 60 days. **b** Percentage of E7-specific and the percentage of EM-like cells within the E7-specific CD8^+^ T cells of the vaccinated mice. **c** and **d** TC-1 tumor outgrowth (**c**) and survival (**d**) graphs of mice immunized with MCMV-IE2-E7 (schematic shown in **a**). The number of tumor-free/total mice is indicated above each tumor out growth graph in (**c**). Tumor out growth was followed for 60 days. The number of tumor-free/total mice is indicated. *, *P* < 0.05; **, *P* < 0.01; ***, *P* < 0.001

To substantiate whether the magnitude of the circulating vaccine-specific CD8^+^ T cell response relates to tumor protection, we IP and SC immunized mice with low inoculum dosages to achieve responses below the 0.3% threshold (Fig. 5a). Infection with a high dose of MCMV-IE2-E7 via the IP and SC route induced as expected stronger CD8^+^ T cell responses (> 0.3%) against the E7 epitope compared to inoculum dosages with 1000 fold less virus, which resulted in responses < 0.3% in the blood (Fig. 5b). The higher response in magnitude correlated to a higher percentage of EM-like phenotype within the total Ag-specific CD8^+^ T cell population (Fig. 5b). Importantly, immunization with high dose MCMV-IE2-E7 via the IP and SC route protected mice from tumor outgrowth (Fig. 5c and d, Group 2 and 3) while immunization with the low dose resulted in less protection (Fig. 5c and d, Group 5 and Group 6). In the SC but not in the IP low-dose immunized group some mice were protected despite circulating E7-specific CD8^+^ T cells below the threshold. Furthermore, similar to the data shown in Fig. 3 IN immunization with 1 × 10^5^ PFU MCMV-IE2-E7 elicited a response of ~ 0.3%, and resulted in partial protection (40% survival) (Fig. 5a-d, Group 4). Again, the IN immunized mice that were protected had E7-specific CD8^+^ T cell responses > 0.3% while the unprotected group had responses < 0.3% (data not shown). When a 200 fold lower vaccine dose (500 PFU) for the IN route was used, which elicited a lower response (~ 0.1%), all the mice succumbed to the tumor challenge (Fig. 5a-d, Group 7). Taken together, these data indicate that the magnitude and EM phenotype of the vaccine-induced CD8^+^ T cells are controlled by the route of vaccination and inoculum dosage, and are principal determinants for the efficacy of MCMV-based vaccine vectors against tumors.

### Immunity to re-infection can hamper the formation of vaccine-induced CD8^+^ T cell responses

**Fig. 6:**
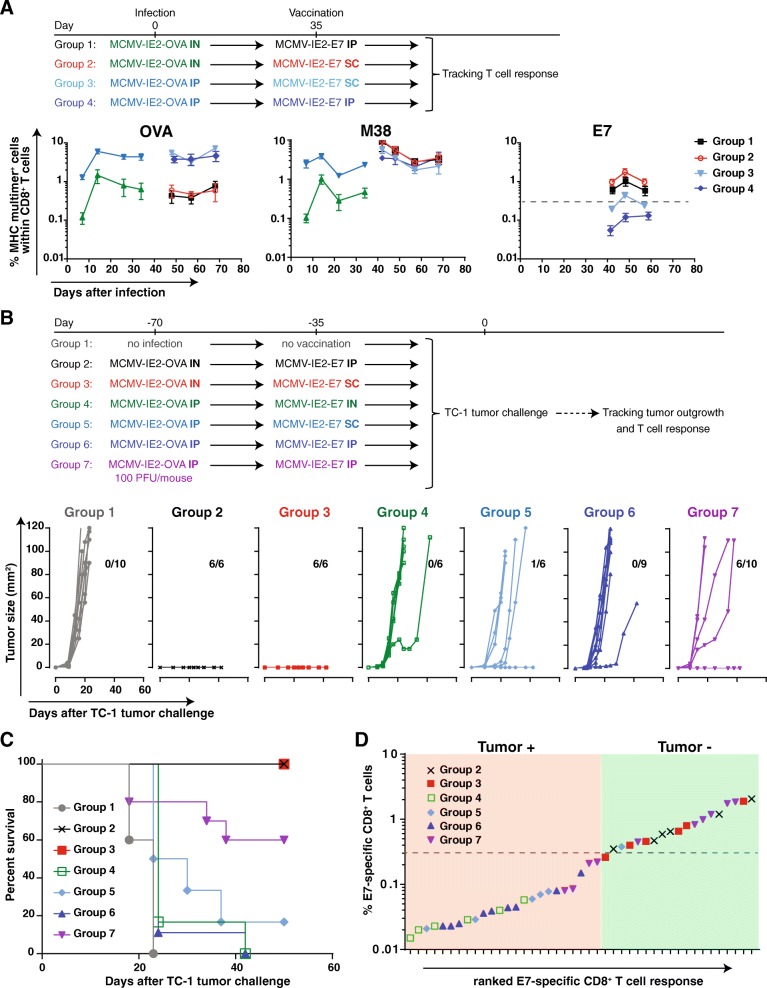
Pre-existing immunity against the MCMV vector affects the strength of T cell responses and anti-tumor efficacy. **a** Percentage of OVA, M38 and E7-specific CD8^+^ T cells, identified using MHC class I multimers. Mice were infected with 1 × 10^5^ PFU via IP or IN MCMV-IE2-OVA at day 0. After 35 days, the mice were vaccinated with 1 × 10^5^ PFU MCMV-IE2-E7 via IP route or with 5 × 10^5^ MCMV-IE2-E7 via the SC route as shown in the schematic. T cell responses were followed for 70 days in blood. Data represent mean values ± SEM. **b** and **c** Tumor outgrowth (**b**) and survival (**c**) of mice infected with 1 × 10^5^ PFU MCMV-IE2-OVA via IP or IN and vaccinated with 1 × 10^5^ PFU MCMV-IE2-E7 via IP or IN route or with 5 × 10^5^ MCMV-IE2-E7 via SC as shown in the schematic. After 70 days, all the vaccinated and control mice were challenged with 1 × 10^5^ TC-1 tumor cells. The number of tumor-free/total mice is indicated above each tumor outgrowth graph. **d** The percentage of E7-specific CD8^+^ T cells within the total CD8^+^ T cell population in the blood (y-axis) plotted against the E7-specific CD8^+^ T cell response ranked from low to high (x-axis) of the mice that were either tumor-free (tumor-) or tumor positive (tumor+). Data are representative of two independent experiments with similar results

Although CMV has the capacity to re-infect the host despite the presence of CMV-specific T and B cell responses elicited upon primary infection, CMV-specific immunity clearly controls viral pathogenesis of primary and secondary infections as shown by the fact that CMV mainly causes disease in immature or immunocompromised situations, and pre-existing immunity strongly limits viremia upon secondary infection [11, 12]. To address the impact of pre-existing MCMV immunity on vaccine-induced CD8^+^ T cell responses and hence the induction of protective immunity, we examined the kinetics of the MCMV vaccine-specific T cell responses in different settings of pre-existing immunity. To mimic the condition of pre-existing immunity in the TC-1 tumor challenge setting, we infected mice first with MCMV-IE2-OVA, which provokes pre-existing immunity but does not elicit tumor-specific T cell immunity. After the establishment of pre-existing immunity, mice were vaccinated with MCMV-IE2-E7. This experimental setup allowed us to follow the antigen-specific T cell response following the first infection and after the subsequent immunization, in which a) the OVA-specific T cell response is primary after the infection and not boosted, b) the M38-specific CD8^+^ T cell response is primary after the infection and then boosted by the immunization, and c) the E7-specific T cell response (which is tumor-specific in case of TC-1 tumor challenge) is not induced during the first infection, hence is primary upon the immunization. Here, we confirmed that IP infection with MCMV-OVA induces higher OVA and M38-specific CD8^+^ T cell responses (~ 5% OVA and ~ 1.5 M38) in blood (Fig. 6a) compared to infection via IN (~ 0.5% OVA and ~ 0.4% M38). Next, we investigated the E7 (tumor)-specific T cell response in these high (IP) and low levels (IN) of pre-existing immunity. Vaccination with MCMV-IE2-E7 via the IP and SC route boosted the M38-specific T cell response (~ 3% M38 compared to 1.5 and 0.5% after primary infection) and this increase was more pronounced in the mice with low pre-existing immunity compared to high pre-existing immunity. Importantly, a higher E7-specific T cell response was also determined in the mice with low pre-existing immunity (0.7–1% versus 0.3%) (Fig. 6a). The CD8^+^ T cell response against the OVA_257–264_ epitope did not change upon subsequent immunization with MCMV-IE2-E7. Together, these data suggest that super-infection occurs more readily upon primary inoculation via the IN route versus the IP route. Moreover, the level of pre-existing immunity affects the magnitude of the T cell responses elicited to MCMV vectored antigens.

To assess how various levels of pre-existing MCMV immunity influence the tumor protection, mice with low and high pre-existing immunity were challenged with TC-1 tumor cells (Fig. 6b and c). Mice with a high level of pre-existing immunity (infected previously with a high dose MCMV-IE2-OVA via the IP route) and vaccinated IP, SC or IN with MCMV-IE2-E7 were not protected (Fig. 6b and c, Group 4, 5 and 6). This correlated with the elicited E7-specific responses in the blood that were < 0.3%, except for one mouse in group 5 (SC vaccinated) which cleared the tumor and correspondingly had an E7-specific CD8^+^ T cell responses > 0.3% (Fig. 6a and d). Moreover, infection with a 1000 fold lower inoculum dosage of MCMV-IE2-OVA via the IP route followed by IP vaccination with MCMV-IE2-E7 resulted in partial protection (Fig. 6b and c, Group 7). Remarkably, the unprotected mice in this group had E7-specific CD8^+^ T cell responses < 0.3% while all protected mice had responses > 0.3% (Fig. 6d). Moreover, in mice infected with MCMV-IE2-OVA via the IN route and then vaccinated with MCMV-IE2-E7 via the IP and SC route, none of the tumors grew out and the mice remained tumor-free (Fig. 6b and c, Group 2 and 3). Importantly, these mice had E7-specific CD8^+^ T cell responses in the blood > 0.3% (Fig. 6a and d). Taken together, these data indicate that the level of pre-existing immunity can set the protection threshold of vaccine-induced CD8^+^ T cell responses by CMV-based vaccine vectors: a strong T cell response against the vaccine vector elicited during primary infection inversely correlates with the ability of an MCMV-vectored vaccine to elicit protective immunity upon super-infection.

### Single-cycle replication is sufficient for the anti-tumor efficacy of MCMV based vaccine vectors

**Fig. 7:**
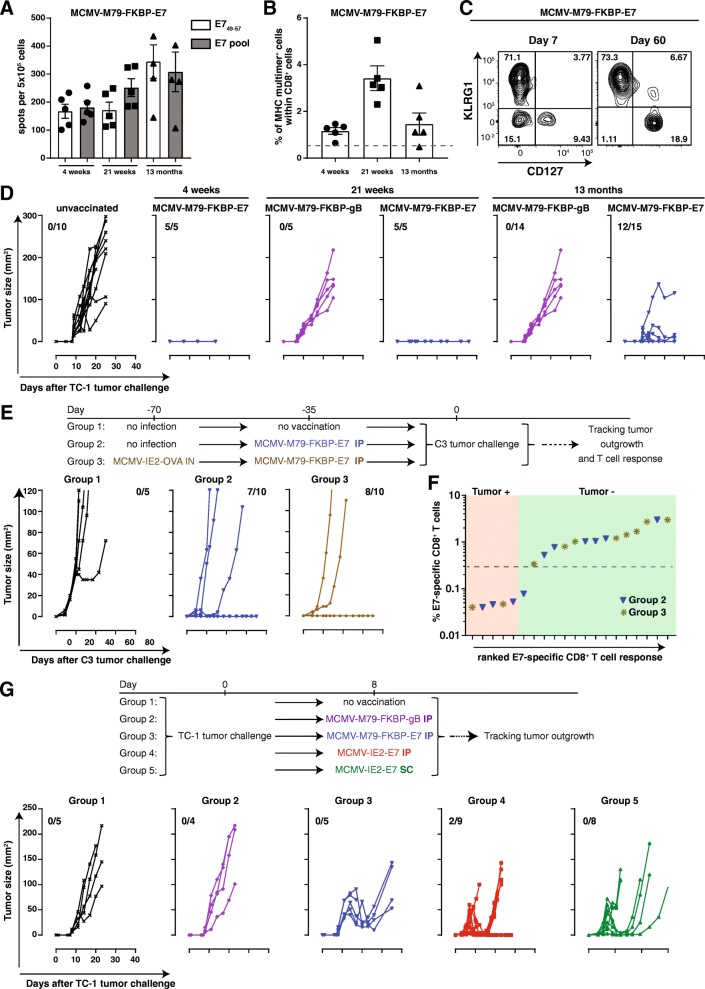
Single cycle replication of MCMV based vector vaccine is sufficient to induce anti-tumor effects in prophylactic and therapeutic settings. **a**. Mice were vaccinated IP with 1 × 10^6^ PFU MCMV-M79-FKBP-E7 or control virus (MCMV-M79-FKBP-gB). Splenocytes were harvested 4, 21 weeks and 13 months after vaccination and stimulated with dominant peptide of E7 or pooled E7 peptides for 36 h (**a**). IFN-γ production was measured by ELISPOT assay. Shown is the number of spots per 5 × 10^5^ cells ± SEM. **b** Frequency of E7_49–57_-specific CD8^+^ T cells within total CD8^+^ T cell population at 4, 21 weeks and 13 months post vaccination. Data represents mean values ± SEM (*n* = 5 mice per group). **c** KLRG1 and CD127 marker expression on E7-specific CD8^+^ T cells at day 7 and 60 post vaccination with MCMV-M79-FKBP-E7. **d** TC-1 tumor outgrowth of the mice that were vaccinated with 1 × 10^6^ PFU MCMV-M79-FKBP-E7 or control virus MCMV-M79-FKBP-gB at 4, 21 weeks and 13 months before tumor challenge. Tumor out growth was followed for 23 or 40 days. **e** and **f** C3 tumor outgrowth of the mice were infected with 1 × 10^5^ PFU MCMV-IE2-OVA via IN route or kept uninfected. After 35 days, the mice were vaccinated with 1 × 10^6^ PFU MCMV-M79-FKBP IP or kept unvaccinated. Mice were challenged with C3 tumor cells after 35 days. Tumor outgrowth was followed for 60 days (**e**). The number of tumor-free mice from the total mice is indicated above each tumor out growth graph. **f** The percentage of E7-specific CD8^+^ T cells within the total CD8^+^ T cell population in the blood (y-axis) plotted against the E7-specific CD8^+^ T cell response ranked from low to high (x-axis) of the mice that were either tumor-free (tumor-) or tumor positive (tumor+) (**f**). **g** Mice were challenged with TC-1 tumor cells, and after 8 days when tumors were palpable mice were treated with 1 × 10^6^ PFU MCMV-m79-FKBP-E7 or control virus MCMV-m79-FKBP-gB via IP, 1 × 10^5^ PFU MCMV-IE2-E7 via IP or 5 × 10^5^ PFU MCMV-IE2-E7 via SC or kept unvaccinated. Tumor out growth was followed for 40 days. The number of tumor-free mice from the total mice is indicated. Data are representative of two independent experiments with similar results

Non-attenuated CMV may cause disease in immunosuppressed individuals. To facilitate the translation of CMV-based vaccine vectors into the clinic, we generated an attenuated E7-epitope-expressing MCMV vector by fusing the FKBP-degradation domain to the essential M79 protein (MCMV-M79-FKBP-E7), which restricts viral replication to a single-cycle in absence of the stabilizing ligand Shield-1 similar to corresponding HCMV FKBP constructs targeting the homologous UL79 gene (Additional file 2: Figure S2). Vaccination with the spread-deficient MCMV-M79-FKBP-E7 elicited E7-specific CD8^+^ T cell responses that were > 0.5% of the total CD8^+^ T cell population at 4 weeks and even up to 13 months after vaccination (Fig. 7a and b). In addition, the E7-specific CD8^+^ T cells displayed an EM phenotype at late time-points post immunization (Fig. 7c). IP immunization with MCMV-M79-FKBP-E7, but not with a control single-cycle virus expressing the immunodominant HSV gB epitope (MCMV-M79-FKBP-gB), prevented TC-1 tumor progression at 4 weeks, 21 weeks and 13 months post-vaccination indicating long-lasting specific anti-tumor immunity (Fig. 7d). In a similar fashion, MCMV-M79-FKBP-E7 vaccination prevented outgrowth of C3 and TC-1 tumors in most mice (Fig. 7e, Additional file 3: Figure S3A and data not shown). Low level pre-existing immunity elicited via IN infection with MCMV-IE2-OVA did not impact the anti-tumor efficacy of MCMV-M79-FKBP-E7 (Fig. 7e). In mice that succumbed to the tumor challenge, the E7-specific CD8^+^ T cell responses were all < 0.3%, while all (except one) of the protected mice had responses > 0.3% (Fig. 7f).

To investigate the efficacy of MCMV-based vaccine vectors in therapeutic settings, we challenged naïve mice with TC-1 tumor cells followed by vaccination when tumors were palpable. Tumor-bearing mice vaccinated with MCMV-IE2-E7 and with MCMV-M79-FKBP-E7 via the IP and SC route resulted in substantial suppression of tumor outgrowth (Fig. 7g and Additional file 3: Figure S3B). Together these data indicate that a single-cycle MCMV-based vector could offer a safe vaccine platform to induce effective anti-tumor CD8^+^ T cell responses in prophylactic and therapeutic settings.

## Discussion

Here we interrogated whether vaccination thresholds based on the level of tumor-specific T cells elicited by CMV-based vaccines can be sharply defined in order to provide correlates of protection. In addition, we examined the critical factors impacting on the protection threshold and hence the efficacy of CMV-based vaccines against cancer. Fundamentally, we found that CMV-based vaccine vectors have the capacity to provide long-lasting tumor-specific T cell responses that protect against tumors, provided that such responses reach a defined threshold.

CMV-based vectors are thus able to generate defined thresholds of circulating vaccine-specific CD8 T cells that provide long-lasting tumor protection. The factors that determine this threshold such as virus dose, route of vaccination and pre-existing immunity have to be extensively studied to consider the development of effective CMV-based vaccines in cancer patients. CMVs are large viruses (> 230 Kb) encoding more than 170 proteins. Although large viral vaccine vectors eliciting strong T cell responses could possibly lead to competition for antigen-presentation and “space”, which may limit additional T cell responses, this seems not to hamper vaccine-specific T cell responses elicited by CMV. Both in mice and monkeys, CMVs have shown to be very potent inducers of T cell responses to antigens in SIV, Mycobacterium tuberculosis, Ebola [14–18] and several cancers including melanoma, prostate cancer and HPV^+^ cancer (unpublished data Klaus Früh). With respect to space limitation for effector-memory T cells. There is evidence in mice and human that superinfections are able to add more T cells to the memory pool [36, 37]. Although, the percentage of a given T cell population can decrease when more memory T cells are added to the memory pool the absolute numbers of a given T cell population generally does not reduce.

Defined thresholds for protection based on disease-specific T cells have been scarcely reported. Schmidt et al. reported that protection against malaria required a large threshold in memory CD8 T cell frequencies, i.e. > 1% specific T cells of the total CD8^+^ T cell population in blood [38]. Here we found that a threshold of > 0.3% specific blood CD8^+^ T cells links to full tumor protection. The sharp threshold we observed suggest that the elicited tumor-specific T cells have a controlled capacity to kill target cells, which is in line with studies showing that the in vivo killing capacity of CD8^+^ T cells is marked yet restricted [39]. This demarcated threshold is clearly reached upon vaccination with high dose inoculums of MCMV vectors delivered via the IP and SC route, and protects against the development of subcutaneous tumors. On the other hand, immunization via the IN route, considered as a natural route of infection [40], resulted in minor vaccine-specific responses and hence lower protection. However, IN immunization was superior in protection against respiratory pathogens compared to IP immunization due to enhanced induction of tissue-resident memory (TRM) T cells [41]. It is thus conceivable that lung tumors are better controlled following vaccination via the IN route. In line with this are the observations that upon SC immunization some mice (10–30%) are protected despite that the tumor-specific CD8^+^ T cell response in the blood is below the threshold. In these mice, the SC immunization may have led to induction of skin TRM cells, known to be able to provide tumor protection [42].

Besides the quantity it is expected that the phenotype of the elicited cells is of influence for the effectivity. Inflating or indefinitely maintained CMV-elicited T cell populations are characterized by their progressive EM-like phenotype [43, 44] and such responses seem to be qualitatively different from CM T cells with respect to their immediate effector functions and tissue homing properties [45]. These features are important for protection against viruses at their sites of entry or reactivation, and may as well be crucial for clearance of tumors arising e.g. from epithelial or endothelial cells, or metastasis. Here and also reported elsewhere [46] a correlation between the magnitude and phenotype was observed. The combination of phenotype with magnitude provides a better predictor for protective thresholds than either parameter alone.

We found that high levels of pre-existing immunity elicited by SC or IP immunization hamper the development of vaccine-specific T cell responses due to anti-vector immunity thereby reducing the efficacy of MCMV-based vaccine vectors. In contrast, induction of pre-existing immunity by the natural IN route enables sufficient formation of vaccine-specific T cells upon re-inoculation leading to tumor protection. In support of the conclusion that CMVs can overcome natural immunity is also the observation that strains of HCMV have been reported to frequently re-infect seropositive individuals [47, 48]. Moreover, naturally infected seropositive rhesus macaques can be readily re-infected with rhesus CMV (RhCMV) even when given SC at very low dose [49]. Also, RhCMV/SIV vector-immunized monkeys can be repeatedly re-infected via the SC route [50]. In this animal model, the ability to re-infect has been shown to be in part due to the effective inhibition of MHC-I antigen presentation [49]. It may be possible to engineer recombinant HCMV vectors that are able to induce strong and protective immune responses despite pre-existing immunity. For rhesus macaques it was already shown that RhCMV vectors containing SIV antigens provide strong vector-elicited T cell immunity and protection against highly virulent SIV challenge in CMV positive animals [50]. Interestingly, the specific vaccine vectors used in these challenge experiments were highly unusual in their T cell targeting phenotype since they elicited CD8^+^ T cells recognizing epitopes presented by MHC class II and MHC-E molecules [51]. Unconventional T cell priming was in part due to conserved MHC class I evasion mechanisms and in part the result of spontaneous genetic changes that occurred in the vectors due to passaging in fibroblasts. However, fibroblast-adapted HCMV-based vaccines did neither elicit HCMV-typical strong effector memory T cell responses nor were the responses unconventional suggesting that unconventional T cell priming is the result of specific mutations that occurred in fibroblasts-adapted RhCMV, but not HCMV [52]. It remains thus to be determined whether HCMV vectors can be engineered both to overcome strong pre-existing immunity and to elicit the desired CD8 T cell response, be it conventional or unconventional, required for protection against specific infectious diseases or cancer. Alternatively, MCMV-based vectors might be used in the clinic as human cells can be abortively infected, still leading to expression and presentation of virally vectored genes [53, 54].

Synthetic vaccines and other viral vectors (than CMV-based such as recombinant vaccinia virus) eliciting T cell responses to the HPV-16 tumor antigens E6/E7 have also been extensively studied [31, 55–60], and some of these vaccines have also shown effectiveness in the clinic [61–63]. Although long-lasting responses with some of these vaccines were accomplished (especially after boosting), the percentage of circulating HPV-specific CD8^+^ T cell responses seemed to be lesser as compared to the responses elicited by MCMV-IE2-E7 as reported here. Whether CMV-based vaccines are effective in patients still needs to be determined, and to accomplish this additional testing is required. A single high-dose vaccination may already be sufficient given the potential induction of large vaccine-specific CD8^+^ T cell responses. Booster regimens with the same CMV-based vectors in therapeutic settings may not work because of high-levels of pre-existing immunity and/or acquired tumor cell resistance after the initial immunization [3]. Yet given their promising results in experimental models an endeavour to test CMV-vectors in the clinic is worth pursuing.

In conclusion, we show that protective thresholds upon immunization with MCMV vectors can be sharply defined via determination of tumor-specific CD8 T cells in blood, and that the inoculum dosage and route of infection are crucial elements determining the magnitude of these cells. In addition, our study shows the importance of determining the level of pre-existing immunity for CMV-based vaccines and suggests including stratification based on the magnitude of CMV-specific T cell responses in pre-vaccinated individuals. The route and dose of immunization may then even be adjusted to improve the vaccine efficacy. Further studies will be needed with the anticipation to improve the efficacy of CMV-based vaccines.

## Additional files

Additional file 1: **Figure S1.** Immunization with MCMV-IE2-OVA induces vaccine-specific CD8^+^ T cell responses. (A) Mice were infected with 1 × 10^5^ PFU MCMV-IE2-OVA via IP or 5 × 10^5^ via SC_._ Frequency of antigen-specific CD8^+^ T cells for the M45 and M38 epitopes of MCMV and of the inserted OVA antigen were identified using MHC class I multimers. Data represents mean values ± SEM (*n* = 6 mice per group). Data are representative of two independent experiments. (B) MC38-OVA tumor outgrowth of unvaccinated mice and of mice previously vaccinated with MCMV-IE2-OVA via the IP or SC route. Mice were vaccinated with 1 × 10^5^ PFU MCMV-IE2-OVA via IP or 5 × 10^5^ via SC. After 35 days (MCMV-IE2-OVA IP) or 70 days (MCMV-IE2-OVA SC), mice were challenged with 2.5 × 10^5^ MC38-OVA tumor cells. The tumor outgrowth was followed for 100 days. The number of tumor-free/total mice is indicated in each graph. Data are representative of two independent with similar results. (EPS 1218 kb)

Additional file 2: **Figure S2.** Fusion of the FKBP-degradation domain to the essential gene M79 results in a single cycle MCMV. Schematic diagram of the construct. The FKBP degradation domain was fused in frame to the N-terminus of M79 via BAC recombination (MCMV-M79-FKBP-E7). In the presence of the small molecule Shield1 the fusion protein is stabilized and virus production occurs. In the absence of Shield1 M79 is degraded and virus production is halted since M79 is essential for late gene expression. (EPS 1463 kb)

Additional file 3: **Figure S3.** Single cycle replication of MCMV based vectors induces anti-tumor effects. (A) Survival of the mice which were infected with 1 × 10^5^ PFU MCMV-IE2-OVA IN or kept uninfected. After 35 days, the mice were vaccinated with 1 × 10^6^ PFU MCMV-M79-FKBP IP or kept unvaccinated. Mice were challenged with C3 tumor cells after 35 days. Survival was followed for 60 days. (B) Survival of the mice which were challenged with TC-1 tumor cells, and after 8 days when tumors were palpable mice were treated with 1 × 10^6^ PFU MCMV-M79-FKBP-E7 or control virus MCMV-M79-FKBP-gB via IP, 1 × 10^5^ PFU MCMV-IE2-E7 via IP or 5 × 10^5^ PFU MCMV-IE2-E7 via SC or kept unvaccinated. Survival growth was followed for 60 days. *, *P* < 0.05; **, *P* < 0.01; ***, *P* < 0.001. (EPS 1213 kb)

## Abbreviations

ACT: Adoptive cell therapy
CD: Cluster of differentiation
CMV: Cytomegalovirus
DMEM: Dulbecco’s Modified Eagle’s medium
EM: Effector-memory
FBS: Fetal bovine serum
FCS: Fetal calf serum
galK: Galactokinase
HCMV: Human cytomegalovirus
HPV: Human papilloma virus
IFN-γ: Interferon gamma
IMDM: Iscove’s Modified Dulbecco’s Media
IN: Intranasal
IP: Intraperitoneal
IV: Intravenous
IVC: Individually ventilated cages
kan: kanamycin
MCMV: Murine cytomegalovirus
OVA: Ovalbumin
PFU: Plaque-forming unit
PSG: Penicillin, streptomycin, glutamine
RhCMV: Rhesus cytomegalovirus
SC: Subcutaneous
SPF: Specific pathogen-free
TRM: Tissue-resident memory
